# A Case of Waardenburg-Shah Syndrome Type 4 Presenting with Bilateral Homochromatic Blue Irises from Pakistan

**DOI:** 10.7759/cureus.3143

**Published:** 2018-08-14

**Authors:** Maira Nusrat, Muhammad Ali Tariq, Saher Aslam, Ahsan Zil-E-Ali, Marwah Shahid, Shafaq Mahmood

**Affiliations:** 1 University of Health and Sciences, Lahore, PAK; 2 Angiocore Lab, Medstar Washington Hospital Center, Washington, USA; 3 Medicine, Jinnah Hospital Lahore (JHL)/Allama Iqbal Medical College (AIMC), Lahore , PAK; 4 General Surgery, Fatima Memorial Hospital, Lahore, PAK; 5 Developmental Pediatrics, The Children's Hospital, Lahore, PAK; 6 Internal Medicine, Rawalpindi Medical University, Rawalpindi, PAK

**Keywords:** waardenburg syndrome, poliosis, heterochromatic irises, homochromatic irises, hirschsprung disease, genetic disorder, autosomal dominant mutation

## Abstract

Waardenburg syndrome (WS) is a rare genetic disorder. It is caused by multiple mutations affecting the melanocytes, leading to a multitude of skin, hair, and eye symptoms. It is an autosomal dominant disease with four subtypes, each presenting with varying degrees of sensorineural hearing loss along with a constellation of other symptoms. Hirschsprung disease is unique to Waardenburg-Shah syndrome subtype 4 and is not associated with any other subtype. We present a case of this subtype 4 that presented with a bilateral sensorineural hearing loss, mutism, delayed milestones, white forelock, Hirschsprung disease, and bilateral blue homochromatic irises, a finding which is not typical for this subtype. This is the first case of WS with homochromatic irises and the fourth case to be reported from Pakistan.

## Introduction

The history of the disease dates back to 1947 when a geneticist named Petrus J. Waardenburg presented a case of a bald, deaf-mute man with blue eyes, having dystopia punctorum lacrimarum, blepharophimosis, and partial iris atrophy [1]. After observing the same presentation in a few other people, he published a research paper in 1951, describing the features of the disease, which later came to be known as Waardenburg syndrome (WS). It is an autosomal dominant disease with an incidence of one in 40,000 [2]. This heterogeneous disease accounts for more than 2% of cases of congenital deafness [3]. There are four different genetic entities of the disease that are clinically differentiated. Type I is characterized by the presence of dystopia canthorum, sensorineural hearing loss, heterochromia iridis, white forelock, hypopigmentation, and synophrys. Type II has the same features like type I but lacks dystopia canthorum. Type III has hypoplastic muscles and contractures of the upper limbs in addition to type 1 features, and type IV is characterized by type II features and Hirschsprung's disease [4-6]. The genetic mutations responsible for WS include the EDN3, EDNRB, MITF, PAX3, SNAI2, and SOX10 genes. These genes are involved in the formation and development of several types of cells, including pigment-producing cells called melanocytes. Mutations in any of these genes disrupt the normal development of melanocytes, leading to abnormal pigmentation of the skin, hair, and eyes and problems with hearing [6].

We report a case of a young child with WS who presented with the chief complaints of Poliosis, Hirschsprung’s disease, and bilateral homochromatic irises.

## Case presentation

**Figure 1:**
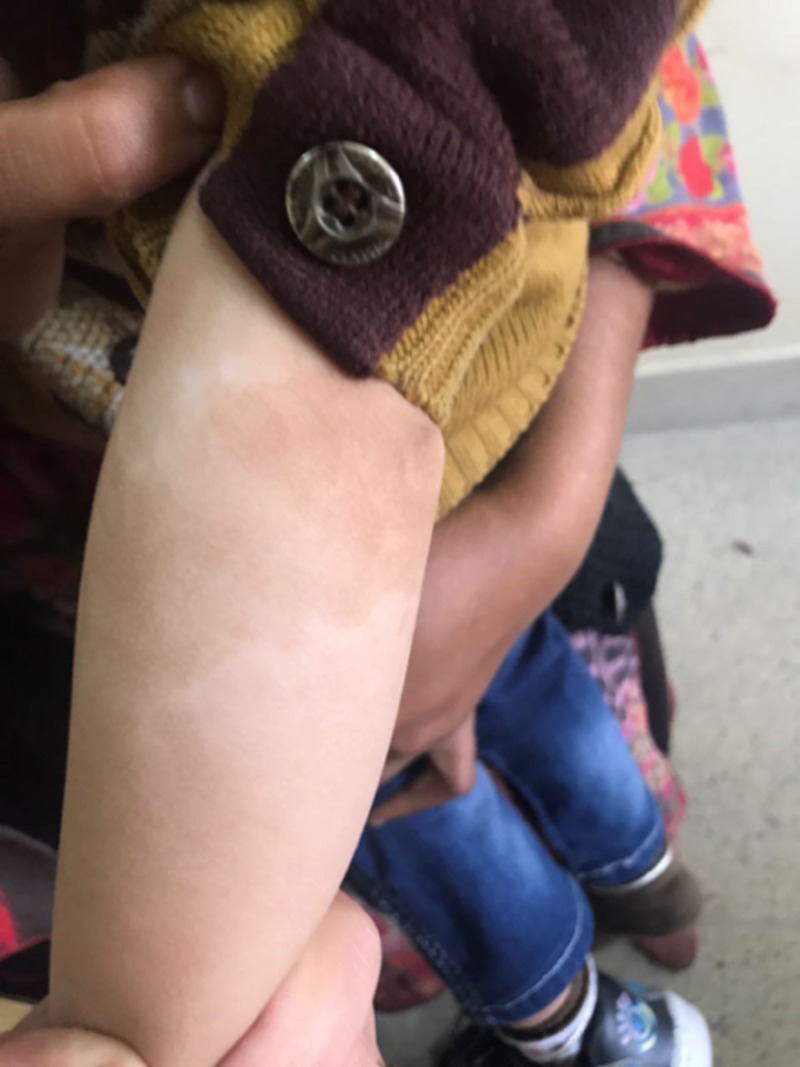
Photograph showing the depigmented patches on the left forearm. (Consent was obtained to publish this image).

**Figure 2:**
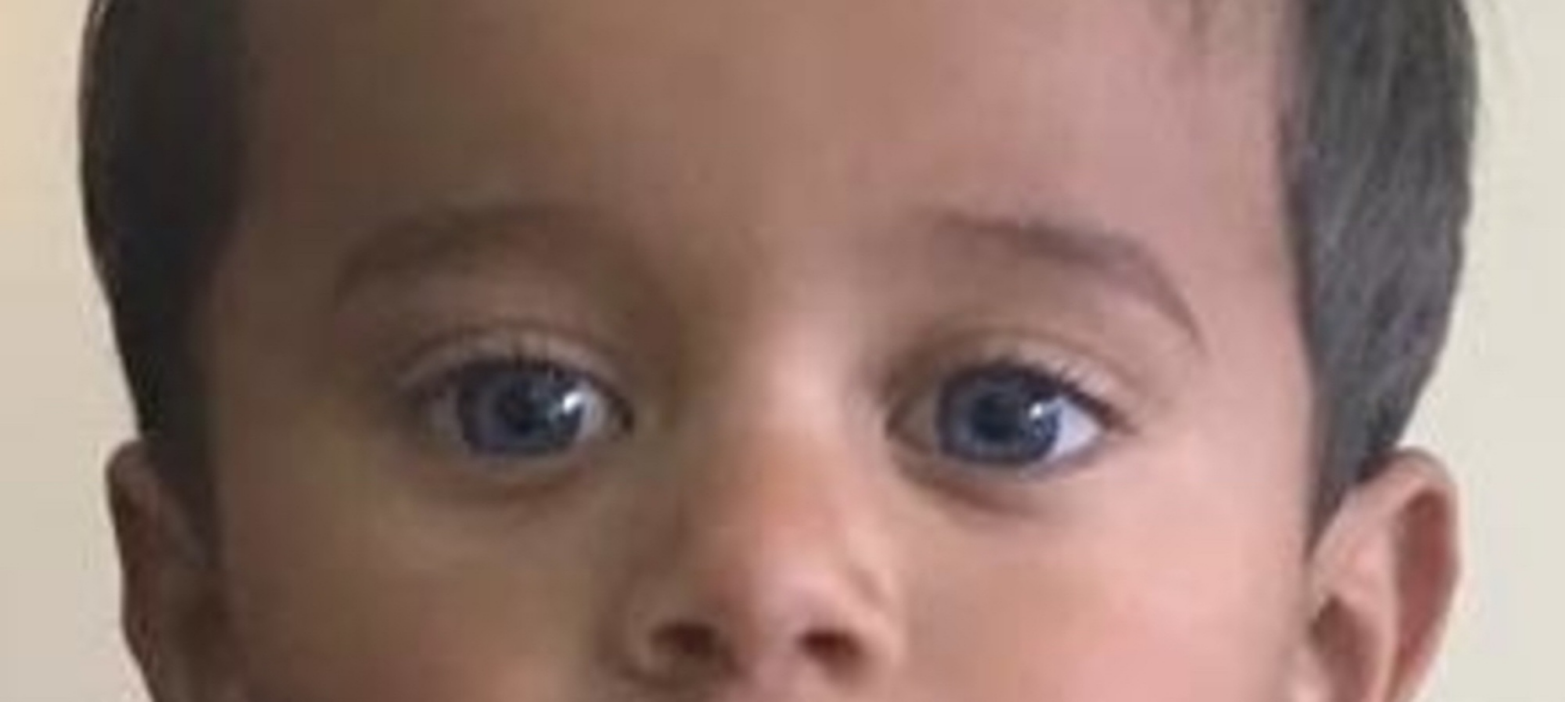
Photograph of the case showing a blue-colored iris bilaterally. Homochromatic irises are not discussed in the literature, although heterochromia is a hallmark of Waardenburg syndrome and its subtypes. (Consent was obtained to publish this image).

A 2.5-year-old male child, apparently healthy but underweight, with a body mass index (BMI) of 17.7 (12.2 pounds, 22.5 inches) presented to the pediatric developmental wellness clinic at The Children’s Hospital, Lahore, Pakistan, with the complaints of complete hearing loss since birth and aphasia. The orientation of the patient could not be assessed due to the aphasia, although he was alert. The patient was afebrile with a heart rate of 85 bpm, blood pressure of 110/85 mmHg, and respiratory rate of 18/min. Upon a physical examination, the patient had blue homochromatic irises with a normal visual response, coarse hair texture, pallor of nails, and a slightly broad high nasal root. Segmental depigmentation was seen affecting the forehead and left forearm (Figures 1-2).

The patient's mother reported the presence of a white hair patch (poliosis) in the frontal hair distribution since birth, which diminished upon cutting the hair. A delay in achieving multiple milestones, including neck holding, crawling, sitting, and walking were also reported. The mother had an uneventful natal history with two healthy daughters without the presence of any similar symptoms in them.

**Figure 3:**
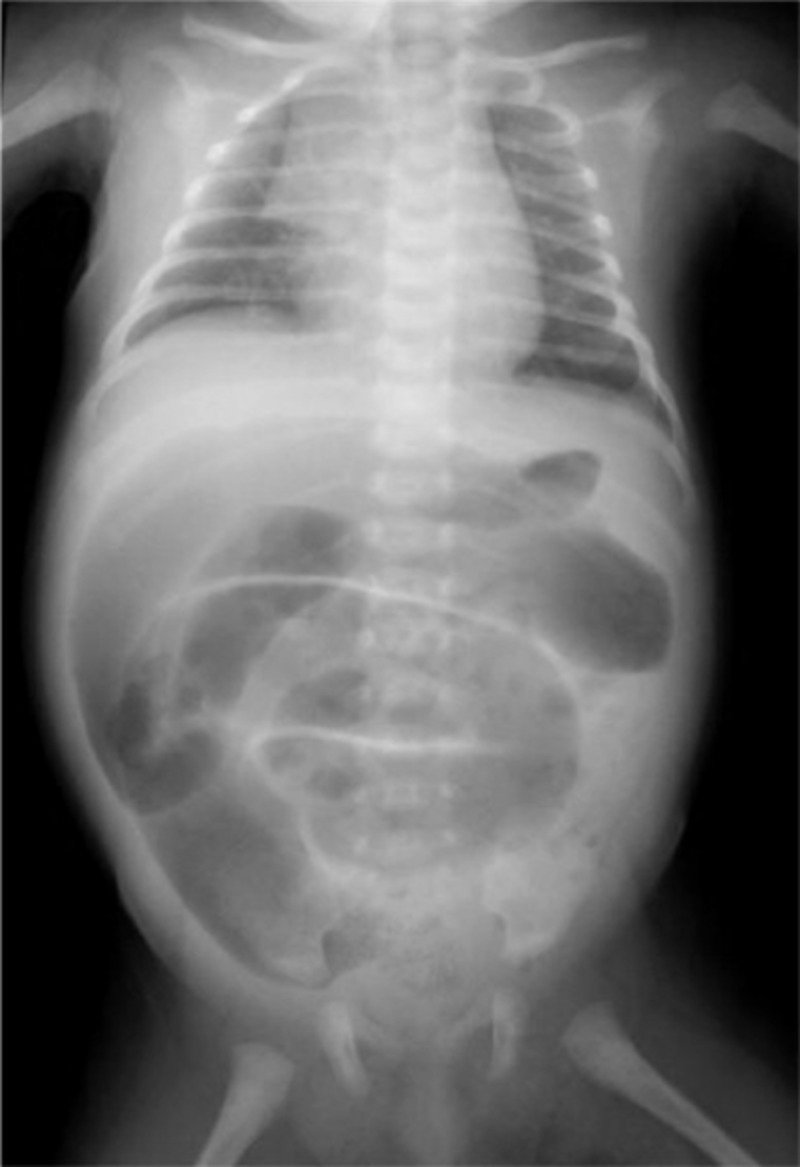
Abdominal plain X-ray shows a generalized distention of the loops of the large intestine. Typical findings of Hirschsprung disease were seen. (Consent was obtained to publish this radiograph).

Past medical history included episodes of bilious vomiting, failure to thrive, and multiple bouts of severe constipation at the age of six months. On imaging, a diagnosis of Hirschsprung's disease was made and confirmed with a rectal biopsy (Figure 3). Later, an end-colostomy at the level of the descending colon was made. The patient had a positive family history of the WS present in the father and paternal aunt but without the history of Hirschsprung’s disease and hearing deficits.

To evaluate the hearing deficit, an auditory brainstem response was done, which showed a bilateral sensorineural hearing loss. The patient also demonstrated complete mutism and lack of response to commands. A DNA sample from the boy was used as a template for a polymerase chain reaction (PCR) to amplify exon 2 of gene SOX10. The resulting product was subsequently sequenced, employing standard methods on an ABI PRISM 377 DNA sequencer. This patient was found to have a novel truncating mutation of the SOX10 gene, on 22q13. The diagnosis of WS-4C was made.

## Discussion

WS is an auditory-pigmentary syndrome, first described in 1951 [7]. It is classified into four subtypes: WS 1-4. Congenital sensorineural hearing loss and pigmentary defects are common to all subtypes. The diagnosis of WS includes both major and minor criteria, as shown in the table below [7-8].

WS-1 requires two major or one major and two minor criteria for the diagnosis. WS-2 is differentiated from WS-1 by the absence of dystopia canthorum (laterally displaced inner canthi). WS-3 presents with musculoskeletal abnormalities (syndactyly, joint contractures, and muscle hypoplasia) in addition to WS-1 features. WS-4 is similar to WS-2 but differentiated by the presence of Hirschsprung disease (Table 1) [9].

**Table 1:** Diagnostic criteria for Waardenburg syndrome.

Major Criteria Congenital sensorineural hearing loss Pigmentary abnormality of the eyes: heterochromia iridis, isohypochromia iridis or pigementary defects of the fundus First degree relative with Waardenburg syndrome Pigmentary defects of hair: white forelock Dystopia canthorum: lateral displacement of inner canthus of eyes (unique to WS1 and 3)
Minor Criteria Leukoderma-hypopigmentation patches on the skin Monobrow/ synophrys Broad nasal bridge Incomplete development of nostrils Prematurely gray hair (under age 30)

**Figure 4:**
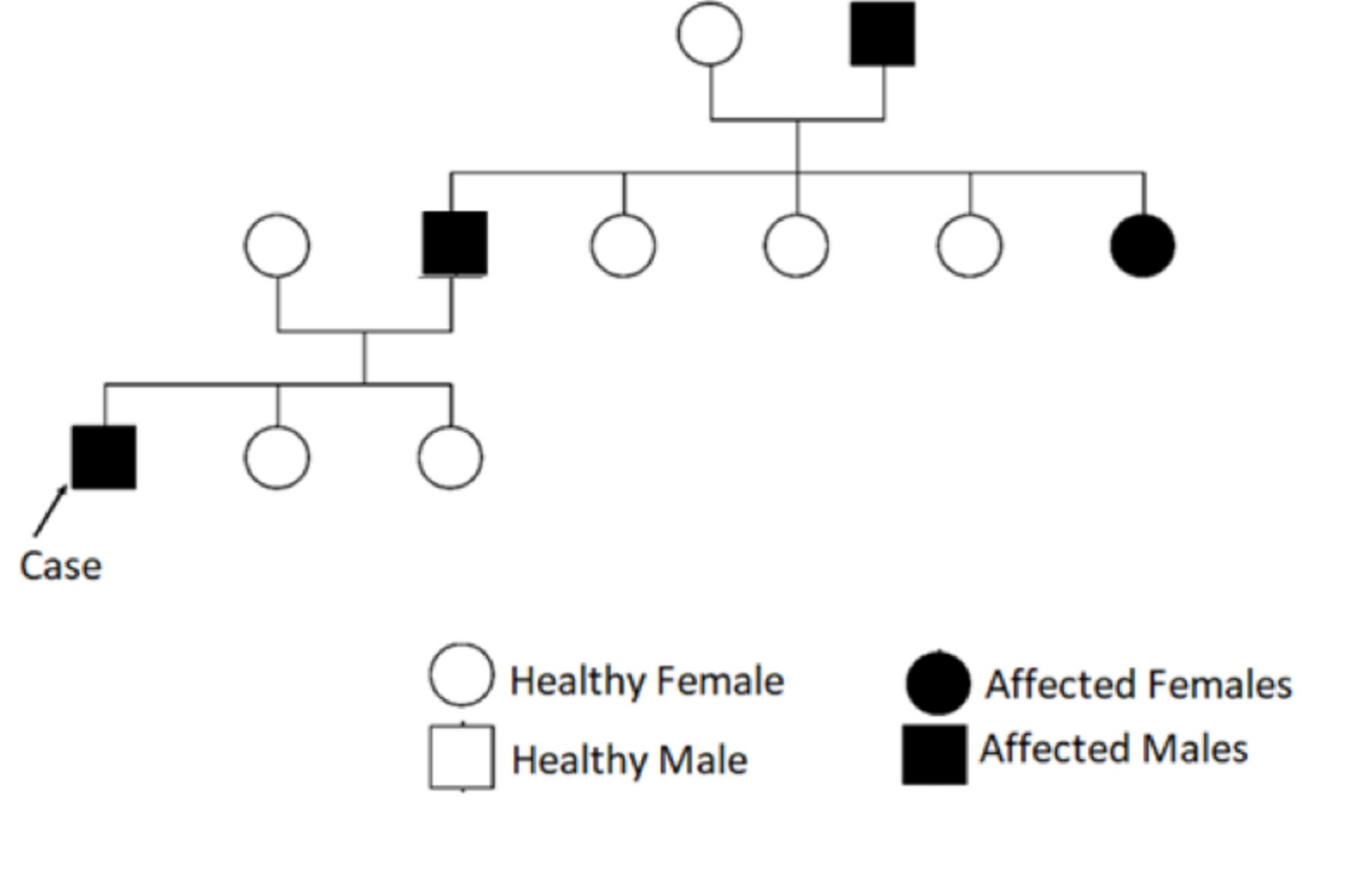
Pedigree of our patient's case, showing Waardenburg syndrome in father and paternal grandfather.

Our patient fulfilled the criteria of the WS-4 syndrome, with the exception of homochromatic blue eyes, which was an additional finding. He presented with delayed milestones, bilateral sensory neural hearing loss, speech deficits, partial albinism, a white forelock, and a history of surgically corrected Hirschsprung disease. On a detailed history and physical examination, a diagnosis of Waardenburg-Shah syndrome was made and was further confirmed by cytogenetics. A history of consanguinity was found in the patient’s family for two consecutive generations. Our patient had multiple family members suffering from similar symptoms of different intensity but Hirschsprung was unique to this child only (Figure 4).

Waardenburg-Shah syndrome(WS-4) is characterized by sensory neural hearing loss, pigmentary defects of the hair and skin, heterochromia of the eyes, and Hirschsprung disease, an intestinal disorder caused by an absence of neural crest cells in the recto-sigmoid region that presents with severe constipation in early childhood [10]. WS-4 can be inherited in both autosomal dominant and recessive patterns [11-12].

WS-4 is a rare type of WS. Only three cases of WS -4 have been reported from Pakistan in the past decade [13]. Consanguinity is common in Pakistan, which may be the underlying cause of rare genetic disorders like WS.

**Figure 5:**
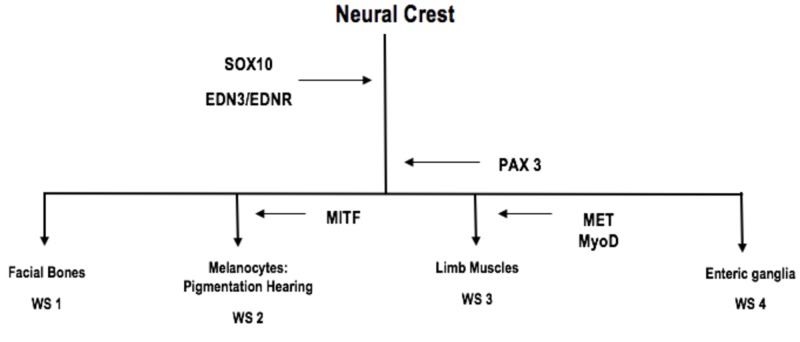
Genes involved in the development of Waardenburg syndrome subtypes and salient clinical features. Our case is of WS4, which presented with a pathology of enteric ganglia, i.e., Hirschsprung disease.

WS-4 is further classified into three subtypes based on the genetic cause. 4A is caused by EDNRB gene mutation; this gene encodes for endothelin receptor type B, a protein involved in signal transduction [14]. This receptor interacts with endothelin proteins and regulates several biological processes, including the formation of blood vessels and the synthesis of hormones. 4B is caused by an EDN3 mutation, which encodes for endothelin-3, a protein involved in the development of neural crest cells, which gives rise to the enteric nervous system and melanocytes. Melanocytes are vital for the pigmentation of skin, hair, and eyes and for the normal functioning of the inner ear. Mutation in the SOX10 gene causes 4C [15]. This gene has a role in the formation of tissues and organs during embryonic development. SOX10 is present in neural crest cells and in the differentiation of neural crest cells into other highly specialized cell types like enteric nerve cells and melanocytes (Figure 5) [15].

Diagnosis of WS can often be missed since all features are not found in every patient. A detailed history, examination, and a high clinical suspicion are required to make an early diagnosis. The prompt institution of medical management can improve the quality of life and the functioning of these patients. Hirschsprung, the hallmark of WS-4, is diagnosed by clinical methods and radiological studies, such as plain abdominal x-ray, barium enema, anorectal manometry, and rectal biopsy. A barium enema can be non-diagnostic in infants with extensive aganglionosis, necessitating a laparotomy for small intestinal biopsy specimens to identify the level of aganglionosis [16]. The full-thickness intestinal biopsy specimens are crucial for making a definitive diagnosis and determine the length of an aganglionic segment.

Surgical treatment depends on the length of the aganglionic segment. For short segments, enterostomy is done initially followed by the Soave endorectal pull-through procedure. The modified extended Duhamel method is employed for long segments The Swenson pull-through and the Kimura-Stringel operation can also be used. Postoperative complications in type IV WS patients are quite similar to the ones seen with short bowel syndromes. Electrolyte imbalance, infections, and total parenteral nutrition (TPN) and catheter-related complications (sepsis and catheter blockage) can develop. The usual cause of mortality in these patients is sepsis and hepatic failure [17-18].

Management is mainly symptomatic. Hearing aids can be employed for hearing deficits and can further help prevent speech and cognitive deficits. Melanin in healthy individuals protects against ultraviolet (UV) damage. As patients with WS have melanin defects, they are advised to use sunglasses and sun protection regularly. Genetic counseling is important for families with multiple family members affected and should be advised to avoid cousin marriages in the future. The parents of our patient were counseled in detail about the etiology and symptoms of WS. The child was referred to a specialist for hearing aid and speech therapy.

## Conclusions

WS-4 is an extremely uncommon disorder, with varying features in every patient. Its diagnosis has always been a challenging task for clinicians due to its rare and nonspecific presentation. Therefore, reporting such cases is of paramount importance to promote early diagnosis and prompt management. We found an unusual sign of homochromatic irises in WS-4, which has not been documented before and needs further exploration. Thus, this finding should also be kept in mind while examining patients presenting with other features of WS-4.
